# A distributed saccade-associated network encodes high velocity conjugate and monocular eye movements in the zebrafish hindbrain

**DOI:** 10.1038/s41598-021-90315-2

**Published:** 2021-06-16

**Authors:** Claire Leyden, Christian Brysch, Aristides B. Arrenberg

**Affiliations:** 1 Werner Reichardt Centre for Integrative Neuroscience and Institute for Neurobiology, University of Tuebingen, 72076 Tuebingen, Germany; 2 Graduate Training Centre of Neuroscience, University of Tuebingen, 72074 Tuebingen, Germany

**Keywords:** Neural circuits, Saccades, Development of the nervous system

## Abstract

Saccades are rapid eye movements that redirect gaze. Their magnitudes and directions are tightly controlled by the oculomotor system, which is capable of generating conjugate, monocular, convergent and divergent saccades. Recent studies suggest a mainly monocular control of saccades in mammals, although the development of binocular control and the interaction of different functional populations is less well understood. For zebrafish, a well-established model in sensorimotor research, the nature of binocular control in this key oculomotor behavior is unknown. Here, we use the optokinetic response and calcium imaging to characterize how the developing zebrafish oculomotor system encodes the diverse repertoire of saccades. We find that neurons with phasic saccade-associated activity (putative burst neurons) are most frequent in dorsal regions of the hindbrain and show elements of both monocular and binocular encoding, revealing a mix of the response types originally hypothesized by Helmholtz and Hering. Additionally, we observed a certain degree of behavior-specific recruitment in individual neurons. Surprisingly, calcium activity is only weakly tuned to saccade size. Instead, saccade size is apparently controlled by a push–pull mechanism of opposing burst neuron populations. Our study reveals the basic layout of a developing vertebrate saccade system and provides a perspective into the evolution of the oculomotor system.

## Introduction

Bilateral coordination of muscle control is a necessary feature of behavior in all bilaterally symmetric animals. As humans, we alternate the movements of our limbs between bilateral (both in-phase and anti-phase) and unilateral states throughout the day^1^. In contrast, our eyes appear to be firmly yoked to each other, pulled apart only through experimental strategies such as Mueller’s paradigm^2^. Yet, during REM sleep the eyes can move independently, performing conjugate, monocular, and vergence eye movements, capable even of moving along orthogonal axes^3^. The ability of the oculomotor system to generate two such behaviorally distinct states, one characterized by bilaterally coupled gaze interspersed with fast reorienting conjugate eye movements (saccades), the other by uniocular independence, provides hints towards the underlying circuits, which are not yet fully understood.

**Figure 1:**
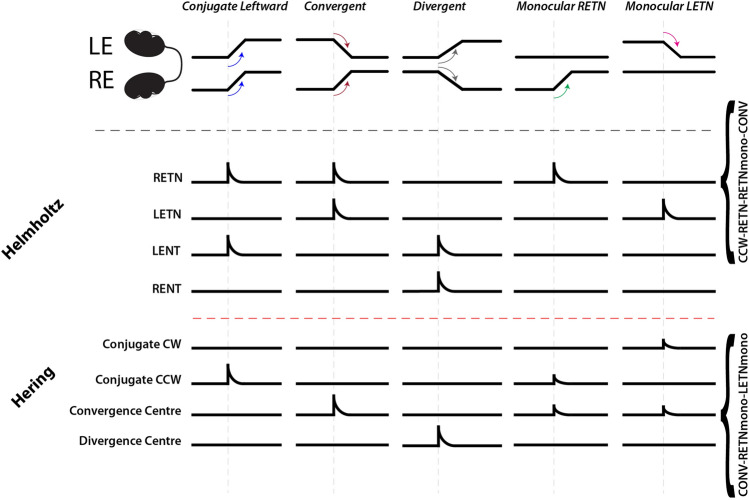
Schematic illustrating the burst neuron populations expected for neurons conforming to either the Hering or Helmholtz hypothesis, and their activity patterns. The descriptors along the right margin indicate how RETN neurons (Helmholtz) and Convergence Centre neurons (Hering) would be classified following our analysis (cf. Table S1). *RE/LE* right eye/left eye; *TN* temporal-nasal, *NT* nasal-temporal.

Saccades are high velocity eye movements which are driven by excitatory burst neurons (EBNs) in the brainstem^4^. The majority of saccades are conjugate, and it is therefore difficult to determine whether they are executed via a single neural command sent to both eyes (argued by Ewald Hering and known as “Hering’s Law of equal innervation”), or via two individual commands defined by separate systems but performed synchronously (suggested by Hermann von Helmholtz). This debate has provided a framework for much of the research into conjugate and vergence eye movements in monkey (reviewed in^5–7^). In monkey, this dichotomy has not been completely resolved, though a Helmholtzian model has recently come into favor^8–10^. Importantly, 80% of EBNs^11^ and the majority of abducens motoneurons^12^ have been shown to encode saccades in a monocular fashion. In favour of a Heringian model, however, are findings that lesions both of the paramedian pontine reticular formation (PPRF)^13^ and medial longitudinal fasciculus (MLF)^4^, can disrupt conjugate but not convergent gaze, and the existence of a vergence center in the midbrain^14^. A key strategy throughout these experiments has been the characterization of neuronal firing during conjugate versus disconjugate saccades. A schematic illustrating the burst neuron populations required for each hypothesis, and how their expected response profiles differ for different saccade types is shown in Fig. 1.

Larval zebrafish are a promising vertebrate model to investigate the development of bilateral oculomotor control, because the activity of complete brain areas can be recorded at cellular resolution in the developing animal^15^. Though the primate system is more complex than that seen in teleost fish, common structures and principles have been shown to underlie the generation of eye movements and may provide insights into an evolutionarily conserved common circuit motif. Recently, Brysch, Leyden and Arrenberg^16^ showed that for low velocity (slow phase) and position encoding neurons, the zebrafish oculomotor system appears to be overwhelmingly monocular, though a large population of binocular neurons (28.6%) was also found. Similarly, Debowy and Baker^17^ found that the adult goldfish velocity-to-position neural integrator for horizontal eye movements contained both monocular and conjugate neurons.

Recent studies have started to characterize the zebrafish saccadic burst generator. The first indications of its location were uncovered by Schoonheim, Arrenberg and colleagues^18^, who were able to elicit saccades via stimulation of neurons in the vicinity of hindbrain rhombomere 5 (also shown by Wolf and Dubreuil^19^). Ramirez and Aksay^20^ reported three distinct types of saccade-related neurons: neurons which fire tonically after saccades, neurons which fire a burst at saccade onset, and ramping neurons which start increasing their firing rate a few seconds before a saccade. These ramping neurons have been hypothesized to be responsible for saccade initiation, by a mechanism possibly involving the anterior rhombencephalic turning region (ARTR) and a neuronal population in rhombomere 7^19,21^.

Our study helps to fill the gap in knowledge that currently exists between command structures and motor output by revealing the nature of bilateral control of saccades. We investigated whether horizontal saccades are encoded binocularly (via summation of conjugate and vergence commands) or monocularly (via independent commands), and how saccade size is determined. We identified putative burst neurons in the hindbrain and found that neural recruitment was biased towards certain saccade types for each neuron, suggesting a certain level of binocular and monocular saccade-type specificity across putative burst neuron populations, which is neither in line with Helmholtz’s nor Hering’s hypotheses. Furthermore, these neurons were also active during small-size saccades in the OFF direction and appeared only weakly tuned to saccade magnitude, suggesting that saccade size is encoded via a push–pull mechanism between opposing burst neuron populations.

## Results

### Zebrafish larvae are capable of conjugate, monocular and disconjugate saccades

**Figure 2:**
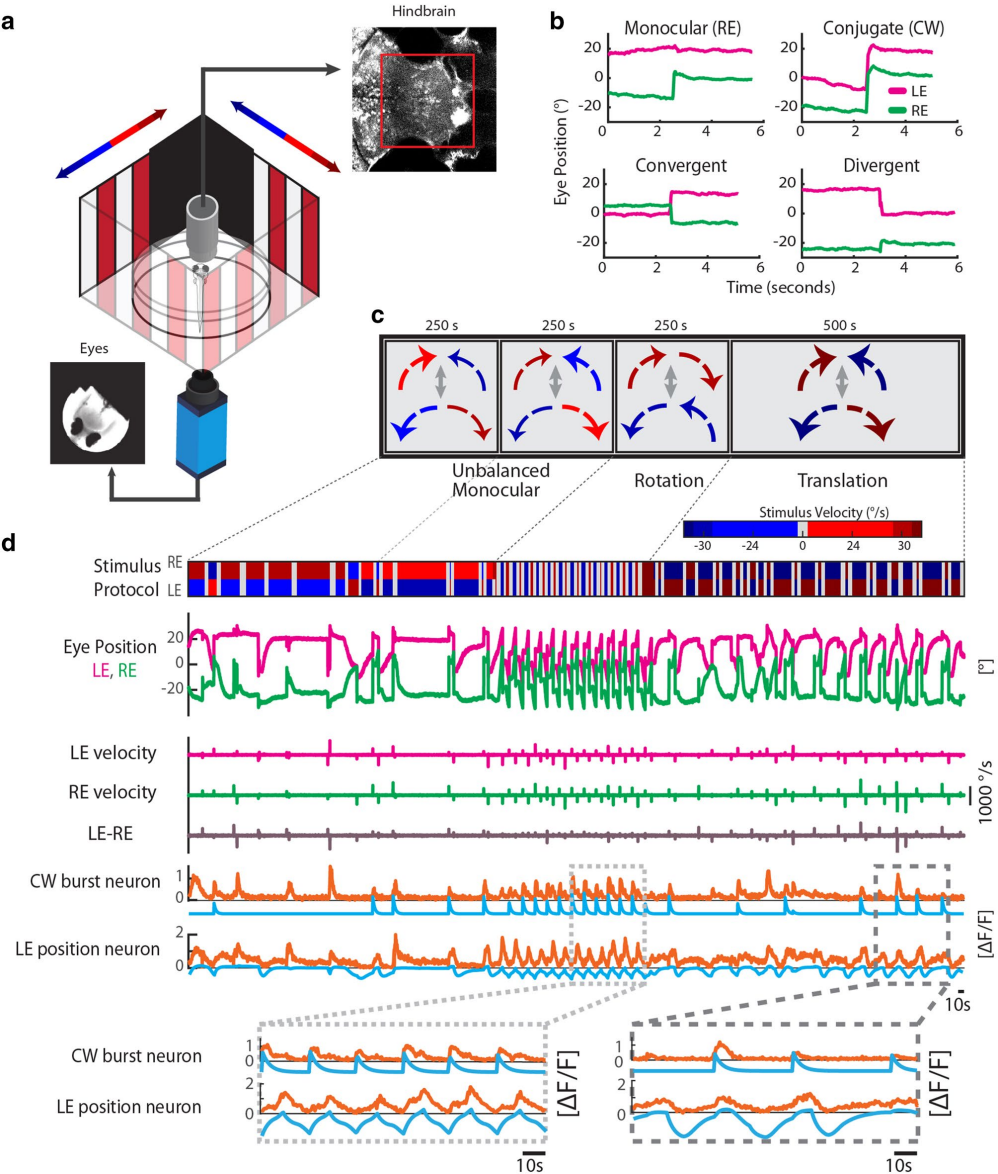
Measurement of cellular calcium signals during quick eye movements. (**a**) Schematic of the experimental setup. Visually evoked eye movements (image of the eyes in the lower left) and cellular GCaMP calcium signals (optical slice of the hindbrain in the upper right) were recorded in agarose-embedded zebrafish larvae. A black foil in front of the area of binocular overlap enabled independent stimulation of the two eyes with differing stimulus speeds. (**b**) Example of various horizontal saccade types: monocular, clockwise (CW), convergent (CONV) and divergent (DIV) saccades. The left eye (LE) is shown in magenta, and the right eye (RE) in green. Positive eye positions refer to rightward eye positions. (**c**) The stimulus protocol consisted of two unbalanced monocular phases, a rotation phase, and finally a translation phase. For each phase, the presented stimulus velocities and directions for the two eyes are illustrated by the size and color of the arrows. During unbalanced stimulation, the absolute stimulus speed was different for the left and the right eyes. For rotation and translation, the absolute speed was the same for the two eyes. (**d**) Example eye positions and saccadic velocities during stimulation are shown in the top four rows. Only saccadic velocities were of interested for this study and therefore slow-phase velocities were not analyzed here (see “Methods”). Note that we paused stimulus motion after each detected saccade (grey shaded area in the stimulus protocol trace) to decorrelate oculomotor position and velocity signals (see “Methods”). Due to the differential stimulation of the left and the right eye, the eye movements were partially unyoked during unbalanced monocular and translation stimulation (see eye velocity difference trace, LE-RE). The calcium activity (orange) of two example neurons is shown in the lower two traces. One neuron is active during CW saccades (CW velocity regressor in light blue), and the other neuron is coding for rightward eye positions (eye position regressor in light blue). The magnified insets at the bottom allow an easier comparison of activity onset relative to eye position change. Though position neurons can be active at saccadic timepoints (e.g. in the right inset), our saccade-related neurons consistently show their highest calcium fluorescence directly after the saccade.

In this study we investigated the monocular and binocular encoding of horizontal saccades within the hindbrain using calcium imaging in semi-restrained zebrafish larvae during optokinetic behavior (Fig. 2a). Since a large fraction of saccades are binocular conjugate eye movements, the naturally occurring correlations of left and right eye movements impede the identification of eye-specific neural activity correlations. We therefore designed a stimulus protocol to experimentally enrich larval zebrafish oculomotor behavior with monocular, convergent, and divergent eye movements (Fig. 2b). This stimulus protocol consisted of a moving bar stimulus being presented to each eye, with alternating phases, during which the velocity of the bar stimulus sometimes differed in magnitude between the two eyes, or had opposite signs (Fig. 2c). This stimulus protocol reliably evoked optokinetic responses, which are characterized by slow velocity eye movements in the direction of stimulus motion. When the eyes reach the periphery, fast resetting eye movements (saccades) occur to enable the next slow phase. For continuous stimulus rotation, this would result in optokinetic nystagmus (repetitions of slow phase and saccade). Calcium signals of oculomotor neurons are difficult to analyze during nystagmus, since the time points of eye position and eye velocity changes are highly correlated. Therefore, to disambiguate eye velocity and eye position related neural activity^16^, we paused stimulus motion for 5–7 s after automatic detection of a saccade and then switched its direction for both eyes (Fig. 2d). Since we focused on neurons active during saccades, we removed neurons from analysis which showed tuning to eye position (two example neurons are shown for comparison in Fig. 2d, see “Methods”).

**Figure 3:**
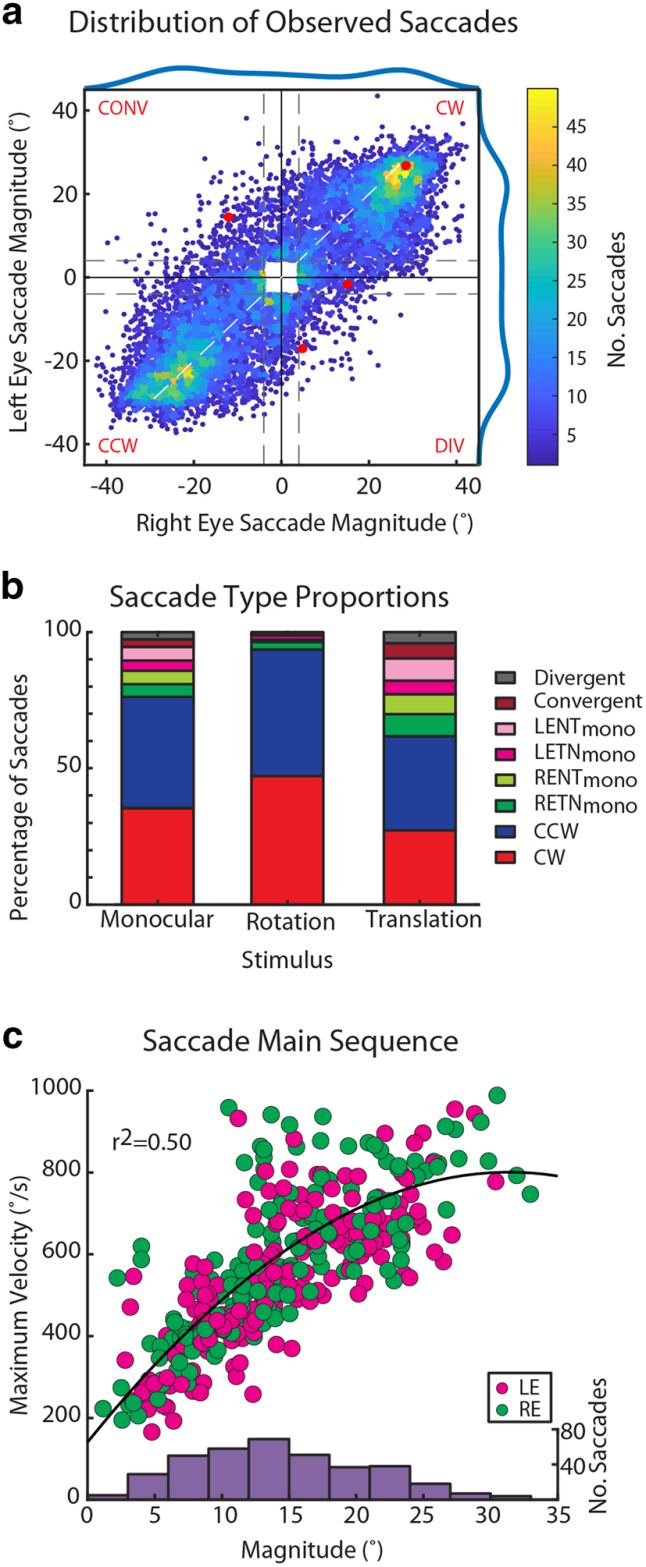
Characterization of binocular saccades. (**a**) Heat scatter plot^48^ showing the distribution of binocular optokinetic saccade magnitudes in all analyzed calcium imaging experiments (n = 30 larvae, 146 recordings, 9,866 data points). The saccades shown in Fig. 2b are plotted in red. Only saccades larger than 4° were included in further analysis for technical reasons. The classification thresholds for monocular, clockwise (CW), counter-clockwise (CCW), convergent (CONV) and divergent (DIV) are indicated as dashed grey lines, and the outer quadrants are labelled in red. (**b**) Proportion of saccade types per protocol phase. Note that the translation and unbalanced monocular stimulus phases elicited higher proportions of disconjugate saccades than the rotation phase. (**c**) Dependence of maximum saccade velocity on saccade magnitude (main sequence). Spontaneous clockwise saccades of both eyes (LE, RE) were recorded at 750 fps (n = 24 zebrafish larvae). A histogram of saccadic velocities is shown in brown on top. The distribution was fitted using a cubic polynomial with a resulting R^2^ value of 0.5. *LETN* left eye temporal nasal; *RENT* right eye nasal temporal; *LENT* left eye nasal temporal; *RETN* right eye temporal nasal; mono: monocular saccade.

Zebrafish performed horizontal saccades with diverse magnitudes and directions in each recording (Fig. 3a). Saccades were categorized based on the direction and conjugacy of both eyes (RE: right eye; LE: left eye). The following eight categories were defined: monocular temporal-nasal (LETN_mono_, RETN_mono_), monocular nasal-temporal (RENT_mono_, LENT_mono_), divergent (DIV), convergent (CONV), clockwise (CW, i.e. rightward), and counter-clockwise (CCW, i.e. leftward) saccades. In the convergent–divergent/translational stimulus phase, 38% of saccades were of disconjugate nature (monocular or vergence eye movements), which represents a six-fold enrichment of disconjugate saccades relative to the observed behavior during the conjugate/rotational stimulus phase (Fig. 3b). Notably, conjugate saccades are still the most frequent saccade type in any of the employed stimulus phases. We did not systematically assess the vertical component of these eye movements, which are small during horizontal stimulation (unpublished observation).

### Spontaneous larval saccades conform to the main sequence

The term main sequence describes the stereotyped relationship between maximal saccadic velocity and saccade magnitude^22^. This relationship has been reported throughout human and monkey saccade literature, and has been used as a tool to identify alterations in saccade kinematics in order to probe the circuitry driving them^23,24^. Separate high-speed recordings of spontaneous saccades in the absence of moving visual stimuli (but in a lit environment) showed that larval zebrafish eye movements also conform to the main sequence (Fig. 3c), reaching speeds of 800°/s for large saccades (for previous reports, see^25,26^). Saccade magnitude and velocity are correlated (R^2^ = 0.5).

### Saccade-associated neurons are preferentially recruited based on the saccade type

To identify whether saccades are driven by mainly monocular coding neurons (Helmholtz’s hypothesis) or binocular coding neurons (Hering’s hypothesis), we labelled each saccade event according to the (mutually exclusive) saccade types (CW, CCW, RETN_mono_, RENT_mono_, LETN_mono_, LENT_mono_, CONV, DIV; 8 in total), and according to the occurring horizontal eye movements of both eyes during the saccade event (RETN, RENT, LETN, LENT; 4 possibilities). Whilst the first eight labels were distinct, the final four labels (RETN, RENT, LETN, LENT) were applied to different combinations of saccade types, e.g. RENT occurs during the CW, RENT_mono_ and divergent saccade types. We reasoned that if the burst system was organized according to Hering’s hypothesis, then we would find individual neurons active during conjugate and monocular saccades, or active during vergence and monocular saccades. In contrast, following Helmholtz’s hypothesis, many neurons should be active for all three types: conjugate, monocular and vergence saccades. These activity combinations are shown in Fig. 1.

**Figure 4:**
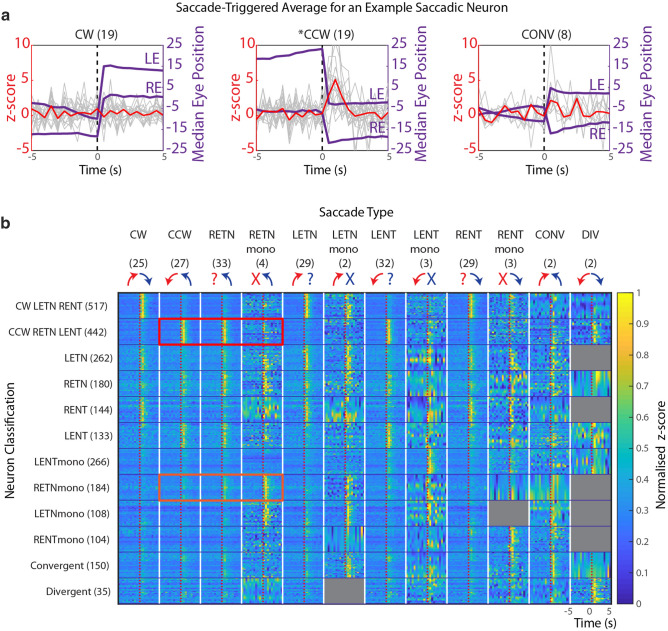
Peri-saccadic activity of different saccade-associated neuron types. (**a**) Saccade-triggered average (STA) of neural activity (calcium signal z-score, red trace). The example neuron was significantly active during or directly after CCW saccades (middle) but inactive during CW or CONV saccades (left and right plots), the full STA for this neuron is shown in Fig. S2. The numbers in brackets indicate the number of saccades in this recording. Median eye positions are plotted in purple. Z-score traces for individual saccades are plotted in grey. The dotted line represents the saccade time point. Saccade-associated neurons were first identified via regressor based analysis, and then classified based on a ranksum test comparing activity in the 9 pre-saccadic sampled time points with that of the saccadic, and two immediate post-saccadic timepoints. Resulting values were Bonferroni corrected (factor of 3). (**b**) Raster plot of neuronal STA activity (normalized per neuron) across different saccades types (columns) and for different neuron types (rows). Neurons were classified according to the STA significance analysis (see “Methods”) and the numbers of identified neurons for each classified neuron type are indicated in brackets. Note that the conjugate CCW-RETN-LENT population is not significantly active during monocular RETN saccades (red rectangle), and the RETN_mono_ population is not significantly active during conjugate CCW saccades (orange rectangle). Within a functional neuron type, each line represents the activity of a single cell. Since certain neuron types occurred less frequently than others, the y-axis has been compressed or stretched to fill each of the squares for visualization purposes. X-axis labels indicate the saccade type, and mean number of saccades of that type occurring per recording. Grey squares correspond to neuron and saccade type combinations for which we had an insufficient amount of data (less than two neurons in the population with 6 saccades of that type).

Using two-photon microscopy and calcium imaging of neuronal somata in the hindbrain, we recorded saccade-related calcium signals at a rate of two frames per second. After semi-automated detection of oculomotor-related neuronal activity (see “Methods”), saccade-triggered average (STA) calcium traces were computed for each neuron (Fig. 4a, Fig. S2). These STA traces show the peri-saccadic activity during the timeframe beginning 5 s before the saccade and ending 5 s after the saccade binned by saccade type; they can be used to distinguish between ramping and bursting neurons as well as position or slow-phase velocity encoding neurons. The individual neuronal STAs were subdivided according to the saccade types introduced in the previous paragraph to identify for which types of saccadic eye movements neural activity was elevated. Since this study focused on neurons with phasic activity during saccades, only neurons with elevated z-scores in the 1.5 s following the saccade were kept, and neurons with preceding high activity levels (e.g. position and slow-phase velocity neurons^16^, or ramping neurons^20^) were excluded from further analysis. A total of 26,220 putative oculomotor-related regions of interest from 30 fish were labelled, and from these 4,372 saccade-associated (SA) neurons were identified. While our recordings should contain neurons shown to be involved in controlling saccades^18^, many of our recorded neurons might carry collateral signals, including those for body movements (see “Discussion”). Furthermore, we did not employ electrophysiology to confirm action potential bursting of our SA neurons.

Our STA-based analysis revealed many functional SA neuron types, the 25 most prominent of which are listed in Table 1 (see Table S2 for a full list of classified types). Most SA neurons were direction-selective and were thus preferentially active for either leftward or rightward saccades. The most frequent functional SA neuron type (517 neurons) was related to binocular counter-clockwise (leftward) saccades and significantly active during counter-clockwise (CCW), RETN and LENT saccades. The second most frequent functional SA neuron type (442 neurons) was the converse, encoding clockwise (CW), LETN, and RENT saccades. This was not surprising, given that conjugate saccades (CCW and CW) were the most frequent saccade types observed and that CCW saccades consist of RETN and LENT eye movements (and CW saccades of RENT and LETN). These two populations conform to aspects of Hering’s hypothesis, although a precise interpretation is impeded by undersampling in our dataset (for many conjugate neurons we don’t know their activity levels during monocular saccades). In ranks 4, 6, 8 and 9 neurons with significant correlation with a single eye were found (LETN, RETN, RENT or LENT, 719 neurons in total), though they did not appear to be related to any particular saccade type. These four neuron types match aspects of Helmholtz’s hypothesis, as these response types encode the movements of a single eye irrespective of the binocular context of the saccadic event. Furthermore, their lack of significant activity during the (frequently occurring) conjugate saccade is in disagreement with Hering’s hypothesis. Unexpectedly, many of the prominent groups of neurons were only active for a single saccade type (saccade type specific), such as monocular saccades (ranks 3, 5, 10, and 12: LENT_mono_, RETN_mono_, LETN_mono_, RENT_mono_, 662 neurons total), convergent saccades (rank 7), or divergent saccades (rank 25). The STA activity of all neurons classified as being related to conjugate, monocular and vergence saccades, as well as those in ranks 4, 6, 8 and 9, is shown in Fig. 4b. We have characterized the populations in terms of which hypothesis they best correspond to in column 4 of Table 1 (see “Discussion” below).

**Table 1:** The 25 most prominent saccade neuron populations found via saccade-triggered average analysis.

Rank	Neuron type	Number of neurons	Hering/Helmholtz/specific
1	‘CW LETN RENT’	517	Hering/undersampled
2	‘CCW RETN LENT’	442	Hering/undersampled
3	‘LENTmono’	266	Saccade type specific
4	‘LETN’	262	Aspects of Helmholtz
5	‘RETNmono’	184	Saccade type specific
6	‘RETN’	180	Aspects of Helmholtz
7	‘convergent’	150	Saccade type specific
8	‘RENT’	144	Aspects of Helmholtz
9	‘LENT’	133	Aspects of Helmholtz
10	‘LETNmono’	108	Saccade type specific
11	‘CCW LENT’	106	Hering/undersampled
12	‘RENTmono’	104	Saccade type specific
13	‘CW LETN’	99	Hering/undersampled
14	‘CW CCW RETN LETN LENT RENT’	98	–
15	‘RETN LETN’	96	–
16	‘RETN RETNmono’	95	Aspects of Helmholtz
17	‘LETN RENT’	93	–
18	‘LETN LETNmono’	92	Aspects of Helmholtz
19	‘CW RENT’	89	Hering/undersampled
20	‘CCW RETN’	84	Hering/undersampled
21	‘CCW’	79	Hering/undersampled
22	‘RETN LENT’	64	–
23	‘RETN LETN RENT’	53	–
24	‘CW’	45	Hering/undersampled
25	‘divergent’	35	Saccade type specific

We were aware of the possibility that the monocular neuron types had been detected by chance given their smaller numbers, the high numbers of recorded burst neurons in total, and the lack of clear anatomical clustering (Fig. S2). However, neurons active during CW or CCW are not significantly active during monocular eye movements (see peri-saccadic activity maps in Fig. 4b), and so the motoneuron drive during monocular eye movements cannot come from these conjugate populations. We thus carried out a permutation analysis to determine whether or not the classification of a small population of the LENT_mono_ functional type could be attributed to chance given the large number of identified ROIs. To test this, we used our algorithm to search for neurons that appear to encode a randomly chosen diverse set of saccades. The probability of identifying a monocular population of this size or larger by chance was (p < 0.0001; comparison to all identified LENT_mono_ encoding neurons). Furthermore, the average anatomical locations of exclusively monocular neurons were both significantly more lateral (permutation test: p = 0.0055, α = 0.0167 (0.05/3)) and also more caudal (permutation test: p = 0.0085, α = 0.0167 (0.05/3)) than those of a randomly chosen selection of identified neurons. Therefore, a small subset of neurons is active exclusively during monocular, but not conjugate movements. The drive during monocular eye movements appears to originate from the combined activity of these monocular-only neurons and Helmholtz-like neurons (ranks 4, 6, 8 and 9), which might form a continuum instead of being clearly separated response types.

**Figure 5:**
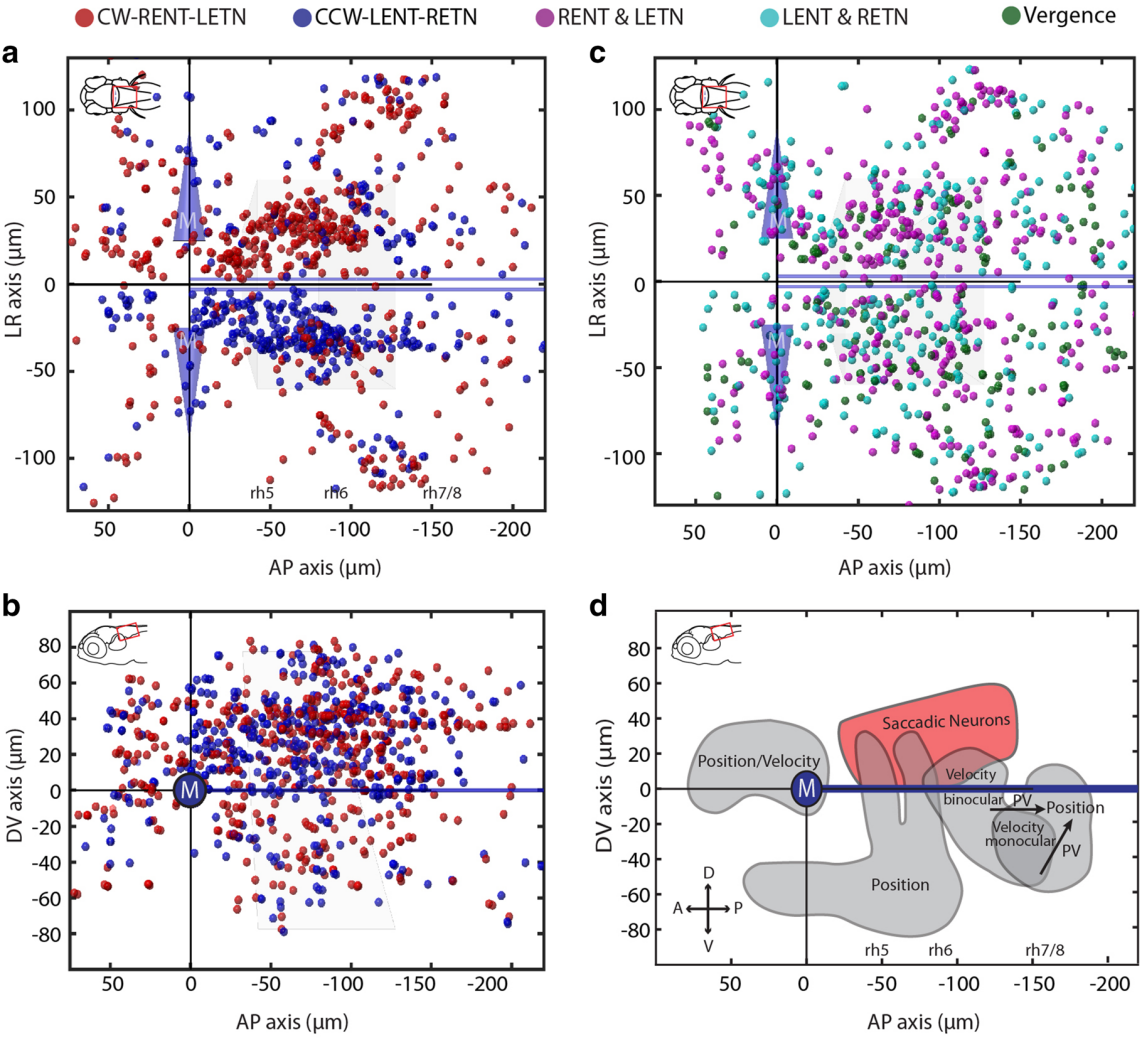
Anatomical distribution of putative burst neurons in the zebrafish hindbrain. Each colored ball represents an identified saccade-associated neuron. (**a**,**b**) Dorsal and sagittal cell maps for CW-LETN-RENT and CCW-LENT-RETN neurons. (**c**) Dorsal cell map for pooled Helmholtz-like neurons representing clockwise (RENT type and LETN type) and counter-clockwise (RETN type and LENT type) directions, as well as vergence (convergent, divergent) neurons. (**d**) Sagittal schematic illustrating the position of the major cluster of SA neurons (red) relative to the position of other neuron types (grey) identified in a previous study (adapted from Brysch, Leyden and Arrenberg^16^, licensed under CC BY 4.0 (http://creativecommons.org/licenses/by/4.0/)). In (**a**–**c**), the trapezoid box represents the abducens nucleus, and the blue cones represent the Mauthner (M) cell bodies. The medial longitudinal fasciculus is represented by the thick blue lines parallel to the larval midline. *D* dorsal; *V* ventral; *A* anterior; *P* posterior; *DV* dorsal–ventral; *AP* anterior–posterior. Data from n = 30 larvae is merged in the anatomical plots (**a**–**c**), corresponding to approximately 3.5–7 completely imaged hindbrains in the range of − 80 to 40 µm (see “Methods”).

In the case of conjugate neurons (CW-LETN-RENT and CCW-LENT-RETN), the neurons appeared to encode both eyes. However, smaller numbers of conjugate neurons with preference for a single eye were found as well (cf. ranks 11, 13, 19–20). To determine the position of the conjugate SA neurons within the zebrafish hindbrain, we generated anatomical maps of the two most frequent SA neuron types (significantly active for conjugate saccades, Fig. 5a,b). The vast majority of SA neurons of the conjugate type are located in rhombomeres 4–6 in the hemisphere ipsilateral to the encoded eye movement direction (ipsiversive), whereas contraversive SA neurons are less frequent and located more caudally (rhombomeres 5–7). In contrast to this lateralization of conjugate-related SA neurons (average lateralization index 0.44), Helmholtz-like monocular neurons (Fig. 5c) were not lateralized to a single hemisphere (average lateralization index: 0.09). Furthermore, within each hemisphere, conjugate-related SA neurons were located more medial (Two sample t-test, p = 4.4 × 10^−11^) more rostral (Two sample t-test, p = 3.6 × 10^−6^) and more dorsal (Two sample t-test, p = 5 × 10^−9^) than Helmholtz-like monocular SA neurons (Fig. 5b,c, Fig. S2). Overall, neurons with saccade-related phasic activity are widely distributed throughout the hindbrain, with clusters of higher density in the more dorsal and medial regions of rhombomeres 4 to 7 (Fig. 5d, Fig. S3).

### Tuning to saccade size: evidence for a push–pull mechanism

**Figure 6:**
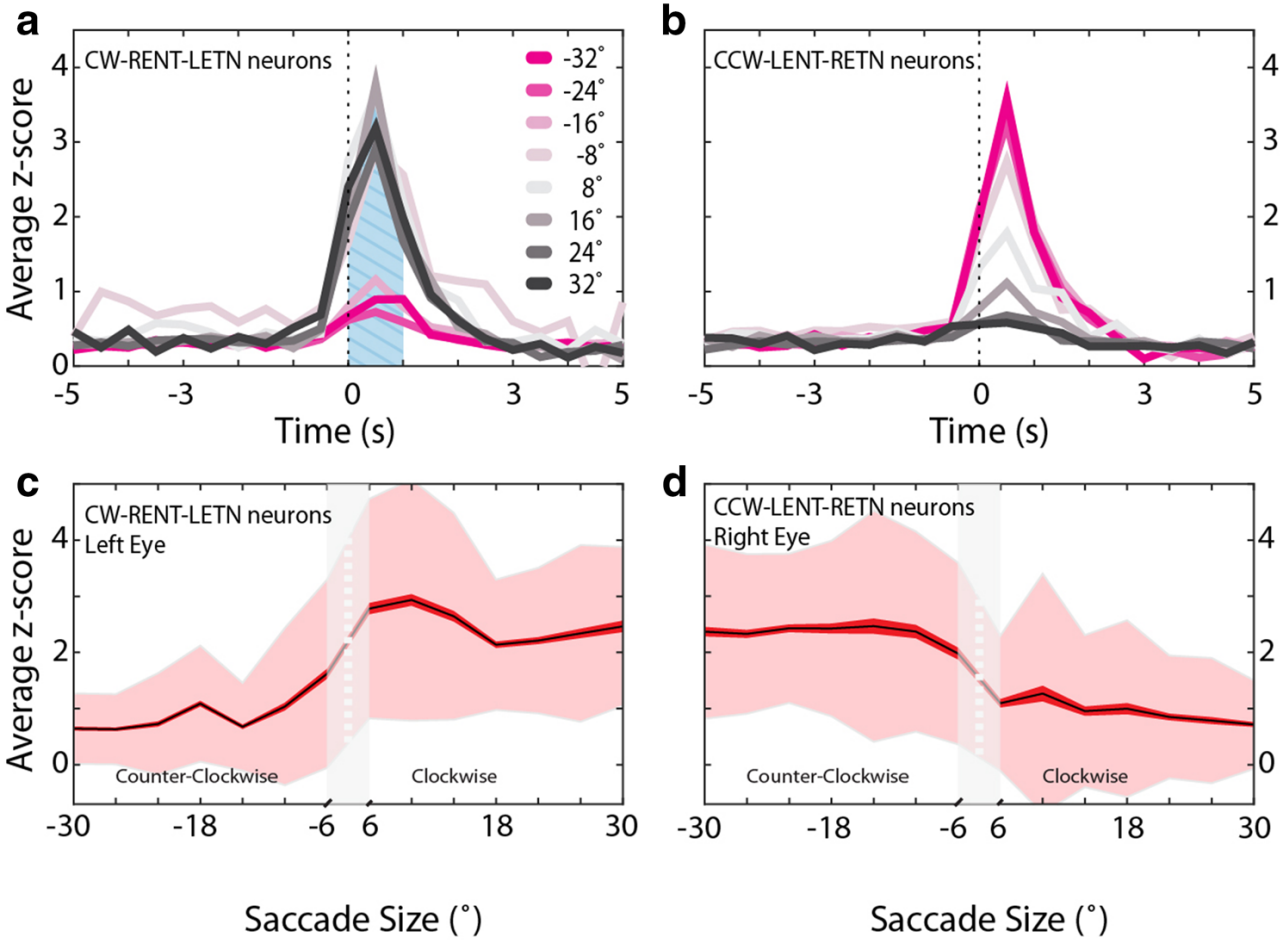
SA neuron tuning to saccade size. (**a**,**b**) Peri-saccadic activity (calcium z-score) of the two major neuron types encoding conjugate saccades, i.e. neuron types CW-LETN-RENT in (**a**) and CCW-LENT-RETN in (**b**). Activity traces have been stratified according to average binocular saccade size in 8° bins (see color legend). The blue hatched area in (**a**) indicate the z-score time points used for the analysis in (**c**,**d**). (**c**,**d**) Separate saccade size tuning curves for the left eye CW (**c**) and right eye CCW (**d**) neurons. Saccade sizes are binned in 4° steps. The black graphs indicate the average z-scores, while red and faint red envelopes indicate the standard error of the mean and the standard deviation, respectively.

Our STA analysis revealed that the majority of neurons were significantly correlated with saccades in one direction only. However, many of the SA neurons also fired during many OFF direction saccades. An analysis of peri-saccadic z-scores according to saccade size and direction showed that the same characteristic activation—albeit at smaller magnitude—was present for saccades in the OFF direction (Fig. 6a,b).

**Figure 7:**
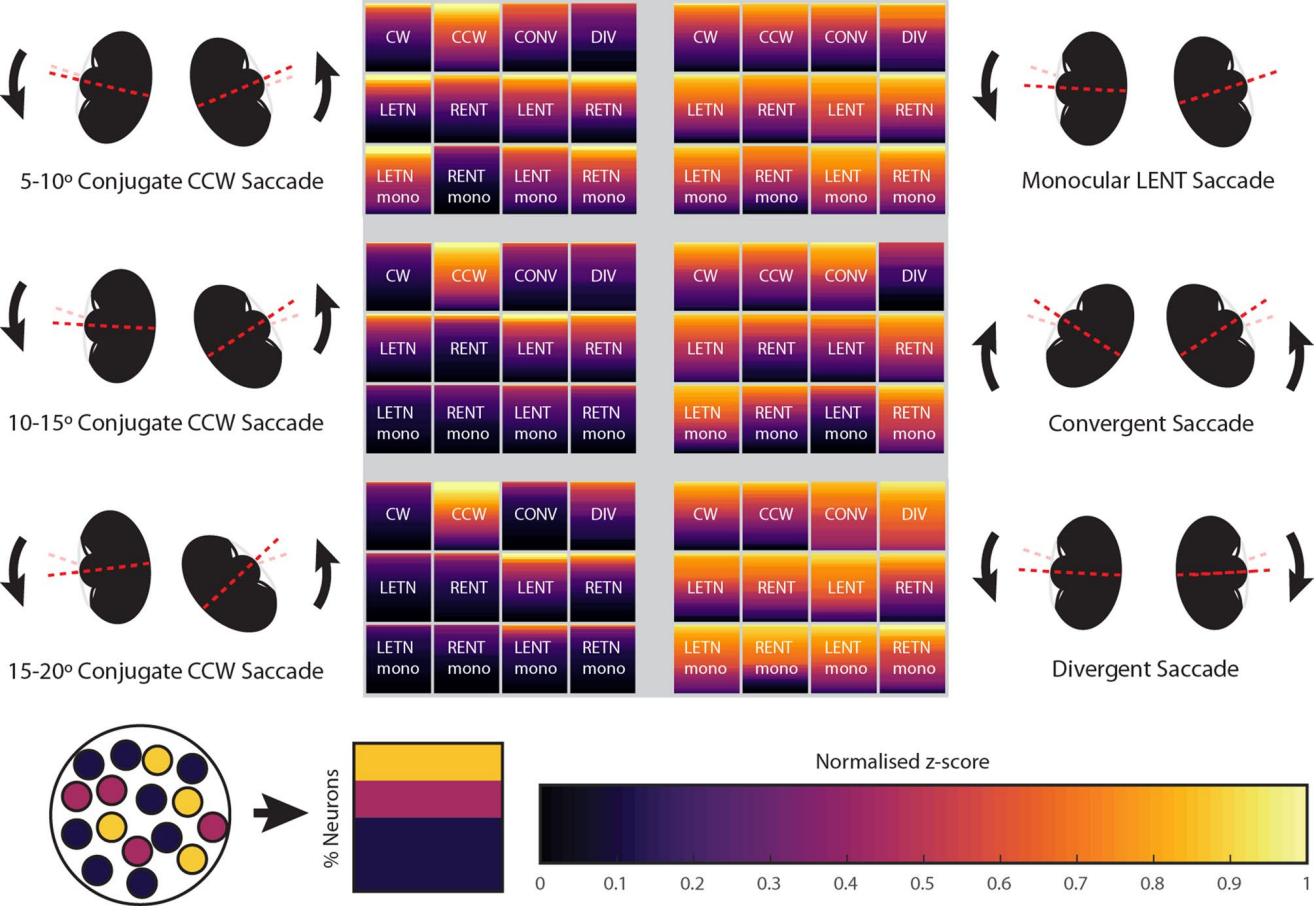
Summary of observed hindbrain saccade-associated activity across different saccade types. Each square refers to the population of neurons listed, except in the case of CW and CCW which refer to CW-LETN-RENT and CCW-LENT-RETN type neurons (see Fig. 4b). For each neuron population and saccade type, the percentage of active neurons and their levels of activity are illustrated by the covered area within the square and the color distribution, respectively (see color legend). The activity of the 12 neuron types is plotted for six different saccade types. Note that many populations are active during small saccades (5°–10° conjugate CCW), even neurons with opposing directional selectivity (e.g. LETN and LENT). However, for large saccades (15°–20° conjugate CCW) mainly the CCW-LENT-RETN population is active, which supports a push–pull encoding of saccade size. *CW* clockwise; *CCW* counter-clockwise; *CONV* convergent; *DIV* divergent; *LE* left eye; *RE* right eye; *NT* nasal-temporal; *TN* temporal-nasal; *mono* monocular eye movements.

In order to understand the relationship between neural activity and saccade magnitude we plotted tuning curves for CW and CCW neurons (i.e. CW-LETN-RENT, and CCW-LENT-RETN). Both groups of neurons were only coarsely tuned to saccade size (Fig. 6c,d). Calcium z-score levels were already high for small-size saccades into the ON direction. To our surprise, z-scores did not increase further for larger saccade magnitudes and instead reached a plateau level. This stagnation of observed calcium indicator fluorescence cannot easily be explained by indicator saturation (see “Discussion”) and suggests that the calcium level or neural activity indeed reached a plateau for large saccade magnitudes. Tuning curves for the LETN, LENT, RETN and RENT neurons followed similar patterns. Since large saccades require more muscle activation than small saccades, and since neither of the tuning curves were consistent with this requirement, this raised the question of how saccades of different magnitude are actually encoded. The non-linearity of the tuning curves suggests that the neurons must interact in some way in order to determine the size. To visualize the predominant SA neuron types and their activity levels, we compared the detected neuronal activity for six characteristic saccade types in Fig. 7. For small saccades (5°–10° conjugate CCW), many functional SA neuron populations are highly active. This includes neurons tuned to the opposite direction and monocular neurons. For large saccades (15°–20° conjugate CCW), most populations are inactive as the saccade is mainly tied to the activity of CCW conjugate SA neurons (significantly active for CCW, LENT, and RETN) as well as Helmholtz-like monocular neurons (LENT and RETN). Contrary to the naïve intuition, the infrequently occurring small-size saccades appear to be driven by the complex interplay of multiple burst neuron types in the zebrafish hindbrain. Therefore, conjugate saccade magnitude appears to be encoded by a combination of size tuning and a push–pull mechanism, in which the balance of neural activity generated by two orthogonally opposing neuronal populations (tuned to CW and CCW saccades) cancels out in order to specify saccade magnitude.

## Discussion

In this study we characterized the saccade-associated network of the larval zebrafish hindbrain by determining the response profile of identified neurons to a range of saccade types. These putative burst neurons showed a clear preference for either leftward or rightward saccades and we observed distinct functional neuron types related to conjugate, monocular, and convergent saccades. Furthermore, we present evidence for a push–pull mechanism specifying conjugate saccade magnitude in combination with saccade size tuning.

### Evidence for Helmholtz-like populations

In other species, especially in macaque monkeys, the existence of monocular versus binocular coding schemes for eye movements has already been studied extensively, with evidence favoring a mostly monocular encoding of burst neurons^9,11,27^. In our neuron type classification scheme, such a Helmholtzian (i.e. monocular, unidirectional) system would rely on four populations (shown in Fig. 1), each active during one of the four possible monocular eye movements and also during corresponding conjugate and disconjugate eye movements, for example a neuron specifically encoding right eye temporal-nasal movements would be classified as type CCW-RETN-RETN_mono_-CONV (first row, Fig. 1). In our analysis these strict Helmholtzian neurons were rarely found (12 total: divided among 4 different classifications), likely because some of the saccade types (e.g. monocular and vergence saccades) were too infrequent to enable a significant result for these saccade types in our STA analysis in any given recording. When inspecting the population activity of the neuron types with ranks 4, 6, 8 and 9 (Fig. 4), it is evident that these neurons are quite similar to Helmholtzian neurons, in terms of their lack of behavioral specificity; we therefore have referred to them as “Helmholtz-like” neurons. These neurons only reach significant STA activity levels, when events from all saccade types (containing the monocular eye movement in question) are pooled. The fact that their activity during conjugate saccades is not significant, an observation which cannot be explained by undersampling, means they are unlikely to be “pure” Helmholtzian neurons. To determine how distinct Helmholtz-like and conjugate populations are, we carried out principal component analysis (PCA) based on their responses to different saccade categories. The PCA and its interpretation is complicated by the scarcity of activity data for certain saccade types. Our analysis of principal components does—however—suggest that the identified response types do not form distinct clusters and instead form continuous distributions (see Fig. S4).

### Evidence for vergence and conjugate neurons

Vergence-associated burst neurons have been found in the brainstem of primates^28–31^, most frequently in the midbrain. These neurons conform to Hering’s hypothesis, according to which a monocular saccade is generated by the combined activity of a vergence and a conjugacy center (where the drive is combined in the moving eye and cancels out for the resting eye). In our classification scheme, we would expect four Heringian populations: a rightward population (CW-LETN-RENT-LETN_mono_-RENT_mono_), a leftward population (CCW-LENT-RETN-LENT_mono_-RETN_mono_), and two vergence populations (LETN_mono_-RETN_mono_-CONV and LENT_mono_-RENT_mono_-DIV) (shown in Fig. 1). For pure conjugate movements only the leftward or the rightward population would be active, for pure convergence only the vergence population, and for monocular movements two populations would be active. The two identified conjugate neuron types (CW-LETN-RENT, CCW-LENT-RETN) closely resemble the pure conjugate Heringian types. Their presence at high numbers suggests a certain degree of binocular saccade encoding, especially since activity during conjugate saccades is smaller for the identified Helmholtz-like neurons. We also detected a sizable population of convergence-specific neurons. However, their inconsistent activity during monocular saccades (Fig. 4) argues against a purely Heringian system. In the macaque, a modulation of vergence burst neuron activity based on the binocular context is also present. A recent study described midbrain burst neurons that are active during disjunctive saccades, but inactive during conjugate or symmetric vergence saccades^32^. In our study, we did not record in the midbrain and further investigation is needed to reveal its role for convergent saccades in zebrafish. Convergent saccades increase the binocular overlap of the visual field, and occur frequently during zebrafish prey capture^33^. As this behavior is important for foraging, and is functionally distinct from optokinetic saccades (in that it does not seek to reduce retinal slip), it is perhaps less surprising that a separate population of convergent-exclusive neurons exists.

### Saccade-type specific neurons challenge both hypotheses

Based on the findings described above, the saccadic control system neither fully conformed to Hering’s nor to Helmholtz’s hypothesis. Instead, the identified neuronal populations showed evidence in favor of both monocular and binocular encoding of saccades. Whilst the majority of identified SA neuron populations were significantly elevated during a particular saccade type or behavior, reminiscent of labelled lines, it is important to note that this should be considered a preferential recruitment rather than true exclusivity. An interesting characteristic of this system is that it suggests burst neuron recruitment is more binocular than monocular in the sense that activity depends on what both eyes are doing during the saccade- a finding which is more in line with a Heringian view.

### Push–pull dynamics may determine saccade size

Previous research in primates has demonstrated that though saccadic neurons are tuned to a specific direction (the ON direction), they are known to still fire a small number of bursts during OFF direction saccades^34^. Cullen and Guitton^35^ systematically analyzed the firing patterns of inhibitory burst neurons and found that half of the neurons recorded had a similar but attenuated rate of firing in the OFF direction. In agreement with these previous studies, our data shows that while zebrafish SA neurons are direction-selective, they also fire at lower magnitude and less reliably during saccades into the OFF direction. Van Gisbergen, Robinson and Gielen^36^ suggested that burst neurons activate motoneurons via a push–pull system, where motoneurons in the right half of the abducens nucleus integrate excitatory and inhibitory burst neuron input from the right and the left hemisphere, respectively. Thus, small-size saccades occur when the bilateral drive generated by the burst system almost completely cancels out at the level of the level of the motoneurons. Our data is in good agreement with such a push–pull system, though we cannot establish the precise temporal relationships as has been identified for different types of primate burst neurons, nor do we have knowledge about the excitatory or inhibitory nature of each recorded neuron. We found that even monocular and Helmholtz-like neurons are recruited during small conjugate saccades (Fig. 7), which is somewhat surprising given that they are not recruited during larger conjugate saccades. Further research is necessary to characterize the interactions between these populations, and determine at what circuit level the observed opposing activity of CW and CCW encoding neurons is integrated.

### Burst neurons were weakly tuned to saccade size

It remains possible that the apparent weak saccade size tuning was due to the calcium imaging approach we used (Fig. 6c,d). Neuronal calcium levels or indicator fluorescence could have saturated for very high bursting activity and could thus have masked a potentially existing strong tuning across the full dynamic range of saccade sizes. While we cannot formally exclude this possibility, we think that this is unlikely to fully explain the measured fluorescence tuning, particularly as the measured fluorescence signals for large clockwise saccades in the range of + 20° to + 32° were significantly smaller than those measured for small saccades into the ON direction (+ 8° to + 20°, left eye p = 4.3 × 10^−6^, right eye p = 2 × 10^−6^). Ramirez and Aksay^20^ describe a network of saccade rise neurons whose activity ramps in advance of saccades. Individual saccade rise neurons are heterogeneously tuned, while the activity of the network can reliably predict saccade onset. A similar population level coding strategy may be used by the saccadic network described here in order to encode saccade size.

### Potential recruitment of vestibular and locomotor systems

When comparing conjugate (left side in Fig. 7) and disconjugate populations (right side in Fig. 7), it is salient that high levels of activity occur during disconjugate eye movements across the identified populations. It is possible that our disconjugate/translational stimuli frequently resulted in the activation of neural circuits involved in locomotion or body posture changes. Therefore, many of our putative burst neurons, especially the disconjugate types, might not be directly related to saccade generation.

We expected the oculomotor circuits to be bilaterally symmetric. However, we observed more conjugate neurons encoding CW saccades than CCW saccades (517 vs. 442 neurons), and more Helmholtz-like neurons encoding left than encoding right eye movements (395 vs. 324). These asymmetric results could potentially be explained by hidden experimental bias (unequal sampling, hardware setup asymmetries). Our analysis also produced many response types for which only very few neurons were identified (Supplementary Table S2). These response types were likely detected at chance or even below chance levels and thus should play a subordinate role in saccadic control. Many of these small-size response populations were not found in every recorded animal, which could have been caused by behavioral bias across recordings and animals, or by true inter-individual circuit differences at this developmental stage.

A previous study suggested that zebrafish burst neurons were located ipsiversively^18^. Indeed, this was the case for the majority of conjugate (but not monocular) neurons found in this study, though some were located contraversively (average lateralization index = 0.44). Generally, our study only revealed correlations and had a low temporal resolution when compared to electrophysiological approaches. It is therefore likely that many of the identified neurons belong to populations performing functions other than facilitating the saccades, as discussed above. Further investigation is needed to identify the burst neurons causally involved in triggering saccades, and to determine their excitatory and inhibitory interactions.

### Monocularity in comparison to other species

Amongst lateral-eyed animals, an ability to uncouple the eyes during slow phase eye movements is not unusual and can be seen in sandlance, pipefish^37^, mantis shrimp^38^, chameleon^39^, and also in goldfish during early development^40^. For goldfish, this ability decreases with time, as shown by Beck, Gilland and colleagues^40^. Much less common, however, is the ability to carry out truly monocular saccades as seen in adult sandlance^37^. The pipefish was shown not to have complete independence, similar to results seen in zebrafish larvae^37,40^, and indeed in our study we could only induce monocular saccades with somewhat unnatural disjunctive stimuli. This raises the question of the ethological relevance of monocular saccades. Adult goldfish have been shown to carry out highly disjunctive saccades whilst swimming, in order to compensate for head turns, and monocular saccades may be an extension of this^41^. As the ability to carry out even slow velocity monocular eye movements decreases as zebrafish develop^40^, it is questionable whether these monocular saccades will remain in adult zebrafish. The possibility that this level of monocularity is a stage in development points towards Helmholtz theory of conjugacy as a learned behavior.

## Conclusion

In conclusion, our characterization of zebrafish burst neuron types starts to reveal the layout of binocular saccadic control in a developing vertebrate. A rich diversity of functional saccade-associated populations are present in the hindbrain and interact in order to generate a wider range of behaviors. Together with the tuning properties of position and low velocity encoding neurons^16^, our findings advance the understanding of larval zebrafish oculomotor behavior at cellular resolution, and suggest coding strategies which may prove to be conserved in other vertebrates.

## Methods

### Zebrafish

Animal experiments and all experimental protocols were approved by the responsible ethics committee of the Regierungspräsidium Tübingen in accordance with German federal law and Baden-Württemberg state law. All animal procedures also conformed to the institutional guidelines of the University of Tübingen. The study was carried out in compliance with the ARRIVE guidelines. 5–7 dpf *Tg(elavl3:nls-GCaMP6s)mpn400* zebrafish larvae were used^42^. All zebrafish used were homozygous for the *mitfa* mutation^43^. Zebrafish were reared in E3 solution at 29 °C on a 14/10 light/dark cycle.

### High-speed camera recordings

High-speed camera recordings were carried out using an iN8-S1 IDT high-speed camera mounted on a stereomicroscope at a frame rate of 750 Hz. Larval zebrafish were embedded in 1.6% agarose; the agarose was then removed from around the eyes to allow unimpeded eye movements. No moving visual stimuli were presented, but the recording chamber (Petri dish lid) was lit. Ten recordings, each 1 s in duration and containing one spontaneous saccade were made of each fish. Recordings were initiated via a motion trigger. Each eye was analyzed separately in the recordings. Saccades were detected when the eye exceeded a velocity of 100°/s for at least 6.67 ms (5 consecutive frames). This step ensured that all included events were saccades; while binocular saccades of sub-threshold velocity did sometimes occur as well (and were excluded from further analysis in the main sequence plot). In line with Gibaldi and Sabatini^44^, the maximum velocity was taken as the highest frame-to-frame velocity. The magnitude of the saccade was taken as the difference between the eye position when the eye began to move in the direction of the saccade, and the eye position when the eyes ceased to move in that direction. Only saccades where data was available for 100 frames before the onset and 500 frames after the onset were analyzed. If more than one saccade was detected in an eye, that individual eye trace was not analyzed. 249 recordings were used, taken from a total of 24 fish: analyzing each eye separately a total of 366 saccades were used to generate the plot.

### Visual stimulation

Experiments were carried out using the animal preparation, optical stimulation setup, and microscope setup described previously^16^, and shown in Fig. 2a. Briefly, larvae were first embedded in 1.6% low melting point agarose in E3. The agarose surrounding both eyes was removed such that both eyes were free to move through a full range of horizontal positions (approximately 30°-40° of dynamical range in each fish). Larvae were then placed under the two-photon microscope. A custom built, 360° surround, square LED arena was used to stimulate the eyes. Previous experiments have elicited disconjugate slow phase eye movements by presenting a moving bar stimulus to one eye, and a stationary stimulus to the other eye: we found that while this stimulus uncoupled slow phase eye movements, the vast majority of the saccades recorded in this condition are conjugate. For this reason, both eyes were provided with moving stimuli throughout the experiment, and uncoupling was achieved by stimuli moving in opposite directions at differing speeds. An opaque black card was also placed directly in front of the arena, at the area of binocular overlap, obscuring an arc of ~ 70°, which allowed both eyes to receive entirely independent stimuli (ignoring potential stimulus reflections). The eyes were then stimulated using a four-stage stimulus protocol, in which the pattern (spatial frequency) of the stimulus was kept the same, but the velocity was altered. In the first two phases the direction and absolute horizontal velocity of the stimulus differed for each eye (unbalanced monocular stimulation). During the third phase the direction and velocity were the same for both eyes (conjugate/rotational stimulus), and during the fourth phase the direction differed but the absolute velocity was the same (convergent-divergent/translational stimulus). The protocol included saccade-triggered motion pauses of 5–7 s followed by stimulus direction reversal. These saccade-triggered stationary periods were used to disentangle position neurons from putative burst neurons. The stimulus presentation lasted 20.8 min per recording and optical plane. Eye position was simultaneously tracked using a CMOS camera (The Imaging Source GmbH, Bremen, Germany) and analyzed using a precursory version of ZebEyeTrack^45^.

Calcium imaging was performed using a MOM microscope (Sutter Instruments, Novato, CA, USA), Coherent Vision-S Ti-Sa laser and a 25 × objective (Nikon CFI75, Tokyo, Japan). Imaging was carried out at a frequency of 2 Hz, a magnification of 1.5x, and at a wavelength of 920 nm for a duration of 2550 frames. For anatomical registration, we used the same registration framework described in Brysch, Leyden and Arrenberg^16^. Since the Mauthner neurons did not express the calcium indicator in our transgenic line, the gap in expression in rhombomere 4 corresponding to their somata was instead used as the registration landmark. Imaging was carried out at 10 µm intervals along the optical axis, and multiple recordings were carried out in each fish in different planes. In the range from -80 µm to 40 µm each dorsal–ventral level within the hindbrain was imaged 7–12 times per 10 µm, a further 4 recordings were made at -90 µm and 50 µm, 3 recordings were made at 60 µm and one recording at 70 µm. In total 146 optical planes were recorded in 30 fish. Imaging was stopped when larvae ceased to reliably perform saccades during the conjugate phase. Since some of the saccade types were infrequent, many individual fish did not show the complete set of observable saccade types in a given recording. For example, divergent saccades were observed in 95 out of 146 recordings, out of which only 6 recordings contained the minimum required amount of 6 saccades. 97 out of 146 recordings were missing one type of saccade, none contained at least 6 of each type of saccade.

### Calcium imaging analysis

All data analysis was performed using custom-written Matlab (The MathWorks Inc., Natick. USA) scripts. Eye traces were correlated with two-photon data in order to find neurons encoding eye movements using a custom Matlab script described previously^46^. High velocity regressors were generated in order to test for correlation^47^. These regressor were based on a velocity threshold of 8°/s (rectified at 8°/s). For convergent and divergent regressors, the regressor was non-zero when the velocity of the slower eye exceeded the 8°/s threshold and eyes moved in opposite directions. Four Helmholtz-type monocular high velocity regressors were generated, one for each eye in each direction. In addition, ten saccade type-specific regressors were generated based on these regressors. These ten regressors were each only active (non-zero entries) for specific conjugate or monocular saccade types. The conjugate regressors were generated separately for each eye, rather than based on averaged eye velocity in order to enable a later test for bias. Once eyetracking data and calcium data were synchronized, saccadic events were detected and a shorter truncated and concatenated calcium signal time series was compiled including all saccadic events and an 8 s window of activity surrounding them (i.e. longer time periods in which no saccades of the chosen regressor types occurred were removed from the trace in order to increase the signal-to-noise ratio of saccade-related signals). This resulted in videos with a minimum of 8.5 s, and a theoretical maximum of the entire recording length, though in practice the true durations were ~ 0.5–7 min in length. The above-described regressors were then generated for these videos and regions of interest were identified based on a pixel-wise correlation with these regressors. ROIs were manually drawn on concatenated videos and then applied to the complete video without truncation (Fig. S5). This process was carried out up to eight times depending on the number of saccade types present in the recording due to the absence of prior knowledge of the recruitment of burst neurons during distinct saccade types. The ROIs were then pooled, and potentially overlapping ROIs were removed based on the distance between centroids. Note that we used the regressors to identify potential neurons and determine saccadic timepoints. However, neurons were ultimately classified based on the results of the saccade triggered average described below.

### Saccade-triggered average (STA) of neuronal activity

Saccade timepoints corresponded to those timepoints in which the eye velocity exceeded 8°/s (as judged from the high velocity regressors). Saccades which overlapped temporally were first removed. An exclusion size of 3 s was used, corresponding approximately to the signal decay kinetics (tau) of the calcium indicator (3.1 s). This step ensured that neural activity could be conclusively correlated with known saccades, and it reduced the risk of misclassification of burst neurons as ramp neurons due to residual activity. 30.6% of the saccadic events were removed from further analysis due to temporal overlap. Each remaining saccade was categorized into one of the 8 unambiguous categories CW/CCW/RENT_mono_/LETN_mono_/RETN_mono_/LENT_mono_/CONV/DIV and in addition the matching ambiguous categories RENT/LETN/RETN/LENT were assigned (see Table S1). For example, each CW saccade was assigned CW, RENT, and LETN. CW and CCW timepoints were taken from the intersection of left and right eye conjugate regressors (i.e., occurrence of binocular conjugate saccade).

For each ROI the 5 s preceding and following a saccadic event were investigated to classify saccade-related neuronal activity. The STA was calculated from the z score of ΔF/F which was first deconvolved using a calcium impulse response function (CIRF) with τ = 3.1 s^47^. The tau value was estimated by fitting an exponential decay function to position correlated neurons. The z-score was calculated via Eq. (1), based on baseline fluorescence activity in the entire recording (upper 20% and lower 5% of activity excluded) as

1 $$\begin{array}{c}z-score=\frac{deconvolved\; DFF-mean\left(baseline\right)}{standard\; deviation\left(baseline\right)} \end{array}$$

For plotting purposes, the z-score was normalized via feature scaling such that all values of each neuron fell in the range between 0 and 1. This was calculated by subtracting the minimum value from all values, and subsequently dividing all values by the difference between the maximum and minimum value. Since this study focuses on burst neurons, we removed neurons from further analysis that carried a strong position signal (position regressor correlation ≥ 0.3). This step excluded 22.1% (5790) of all identified ROIs from further analysis. Rank sum tests were carried out in order to determine whether responses adhered to the expected characteristic burst profile (activity onset at the timepoint of the saccadic event). The baseline was determined for each saccade type from the median calcium signal during eight time points (4 s) preceding the saccade (from 5 s before until 1 s before) and cells were deemed active if the baseline value was significantly different from the signal at the saccadic timepoint, or either of the two points (corresponding to 1 s) following it (to account for slow calcium signal offset kinetics). The rank sum tests had a significance level of 0.05 which was then Bonferroni corrected, i.e. 0.05/3. To exclude potential saccadic ramping neurons from further analysis, the significance for the two timepoints preceding the saccadic timepoint was also tested via rank sum test with Bonferroni correction i.e. α > 0.05/2, (removed 268 ROIs, 1.31% of all remaining neurons). In order to exclude neurons whose activity was decreased rather than increased during saccades, neurons which passed the rank sum test were also excluded if they didn’t meet a threshold z-score response of 3 for 60% of the relevant saccades (removed 14,404 ROIs, 54.9% of all identified neurons). This threshold was chosen based on visual inspection of the data. All 12 categories were analyzed independently, and a total of 4372 remaining neurons with clear saccade-related activity were identified.

### Tuning curves

In order to calculate tuning curves for conjugate SA neurons (significantly active for “CW-LETN-RENT” and “CCW-LENT-RETN”, see “Results”), all neurons were first identified using the STA. For each saccade, and each neuron, the average saccade-related activation magnitude was then calculated as the sum of the z-score for the saccadic timepoint and two following points divided by three. Saccades were then binned in 4° bins based on saccade size, and the mean peri-saccadic (saccadic time point plus the two following time points) calcium signal z-score for each saccade size bin was calculated for each neuron. The population tuning curves were then generated based on the mean across neurons of all individual neurons’ mean z-scores, and plotted alongside the inter-neuronal standard deviation and standard error of the mean.

### Permutation test for monocular neurons

A permutation test was carried out in order to determine whether the incidence of monocular-classified neurons was above chance level. In order to calculate this, only the 26 recordings with enough LENT_mono_ saccades present in order to classify these neurons were used. In these recordings an average of 7.46 (± 1.8 SD) LENT_mono_ saccades occurred. For each saccade event time, the saccade identity of the saccade event was randomly reassigned to one of the known other saccadic timepoints within the recording, to generate a new file where the total number of each type of saccade remained the same but the saccade magnitudes and timepoints were redistributed (permuted). This was done 1000 times, to create 1000 different versions of each recording. The STA analysis was carried out 1000 times on each of the 26 recordings chosen, each time using a randomly generated saccade timepoint file. The number of neurons which passed the LENT_mono_ rank sum test was calculated and compared with that of the true recordings in order to generate a one-tailed p value.

### Permutation test for anatomical centroid locations

A subset of all identified neurons were pooled into four groups: conjugate, Helmholtz-like, monocular and vergence. The conjugate group contained only CW-LETN-RENT and CCW-LENT-RETN neurons, the Helmholtz-like group contained only RENT, LETN, LENT and RETN neurons, the monocular group contained only RENT_mono_, LETN_mono_, LENT_mono_ and RETN_mono_ neurons, and the vergence group contained only “convergent” and “divergent” neurons, other neurons were not used. A permutation test was carried out on the anatomical center-of-masses for each group. For the permutation, we shuffled the neuronal SA type across identified anatomical positions of neurons, but the proportion of occurrence of each SA neuron type corresponded to the proportion in the true recordings. To achieve this, the proportion of each type of neuron (conjugate, Helmholtz-like, monocular, vergence), versus the total number of STA-identified neurons was calculated for each recording (e.g. #mono/#STA-identified neurons), and a matrix of these proportions was generated ([ratio_conj_ ration_Helmholtz_ ratio_mono_ ratio_verg_]* no. of recordings, 4*146). The proportions were rounded downwards so that the sum of proportions could not be greater than 1. This proportions matrix was then shuffled into 10,000 permutations. A vector containing the shuffled SA neuron proportions was generated for each recording. The identified neurons (anatomical positions) were then randomly reassigned to SA neuron types according to the permuted proportions of SA neuron types in each recording. For example, if the fourth row had the entries [20 50 20 10], then in the 4th recording, 20% of the neurons were randomly assigned conjugate identity, 50% Helmholtz-like, 20% monocular, and 10% vergence identity. This yielded 10,000 populations of each of the four groups of neurons. The center-of-masses of all simulated anatomical neuron positions of each iteration were calculated, and compared to the true center-of-masses of the three experimental groups. The resulting p value was multiplied by 2 to account for the two-tailed test. A Bonferroni post correction was then applied to the results to account for the x, y and z comparisons (factor 3).

### Lateralization index

The lateralization index was calculated as the absolute value of the proportion of neurons on one side (hemisphere) of the hindbrain, versus on the other side of the fish. The index ran from 0 to 1, where a result of 0 corresponds to an even distribution of neurons on either side of the fish, and 1 to complete lateralization of the neuronal population.

### Statistical analysis

During the neuron classification steps, 12 independent saccade categories were defined, and analyzed separately. All statistical tests were performed using Matlab. For the saccade-triggered average testing, rank sum tests were carried out using the Matlab command *ranksum*; this test was chosen as the data was non-parametric. Significance was determined at α = 0.05, with Bonferroni correction for multiple comparisons applied to all rank sum tests and bootstrapping. Two-sample t tests were carried out on the tuning curves using the Matlab command *ttest2.*

## Supplementary Information

Supplementary Information.
