# Supplementary material for: A distributed saccade-associated network encodes high velocity conjugate and monocular eye movements in the zebrafish hindbrain
Source: Sci Rep. 2021 Jun 16;11:12644. doi: 10.1038/s41598-021-90315-2 (PMC8209155; doi:10.1038/s41598-021-90315-2)
Supplement: Supplementary file 1 — Supplementary Information. [file 41598_2021_90315_MOESM1_ESM.pdf]

Supplementary Information

Title: A distributed saccade-associated network encodes high velocity conjugate and monocular eye movements in the zebrafish hindbrain

Author names and affiliations: Claire Leyden[1,2], Christian Brysch[1,2], Aristides B. Arrenberg[1*]

[1]Werner Reichardt Centre for Integrative Neuroscience and Institute for Neurobiology, University of Tuebingen, 72076 Tuebingen, Germany

[2]Graduate Training Centre of Neuroscience, University of Tuebingen, 72074 Tuebingen, Germany

Corresponding author email address: *aristides.arrenberg@uni-tuebingen.de

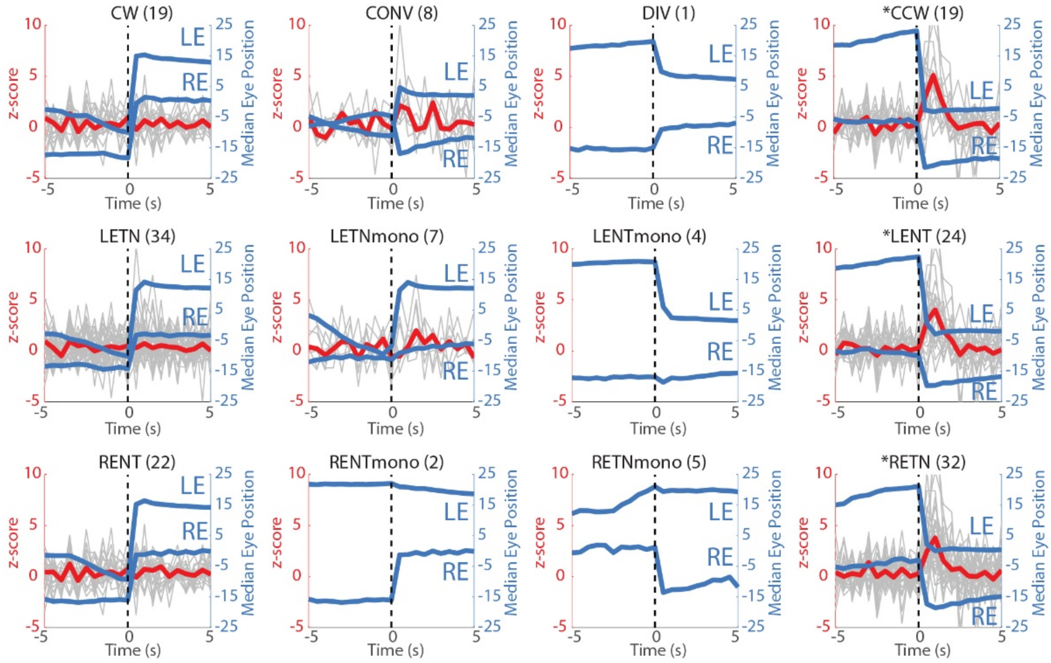

Supplementary Figure S1: Saccade-triggered average analysis. Saccades from individual recordings were assigned to twelve different saccade type bins, and each bin was analyzed independently for each cell. The analysis of one individual cell is shown. Z-score calcium activity traces for individual saccades are shown in grey, with the median trace shown in red. Z-score traces are not shown for bins containing <6 saccades. The median eye position is shown in blue. The dashed black line is the saccadic time point, and activity in the 5 seconds preceding and following the saccade is shown in each plot. * denotes bins where the rank-sum test indicated significant saccade-related z-scores for the saccade type in question. This cell was classified as CCW-LENT-RETN. STA: saccade-triggered average; CCW: counter-clock wise; LETN: left eye temporal nasal; $LETN_{mono}$: left eye temporal nasal monocular; LENT: left eye nasal temporal; $LENT_{mono}$: left eye nasal temporal monocular; RENT: right eye nasal temporal; $RENT_{mono}$: right eye nasal temporal; $RETN_{mono}$: right eye temporal nasal monocular; RETN: right eye temporal nasal.

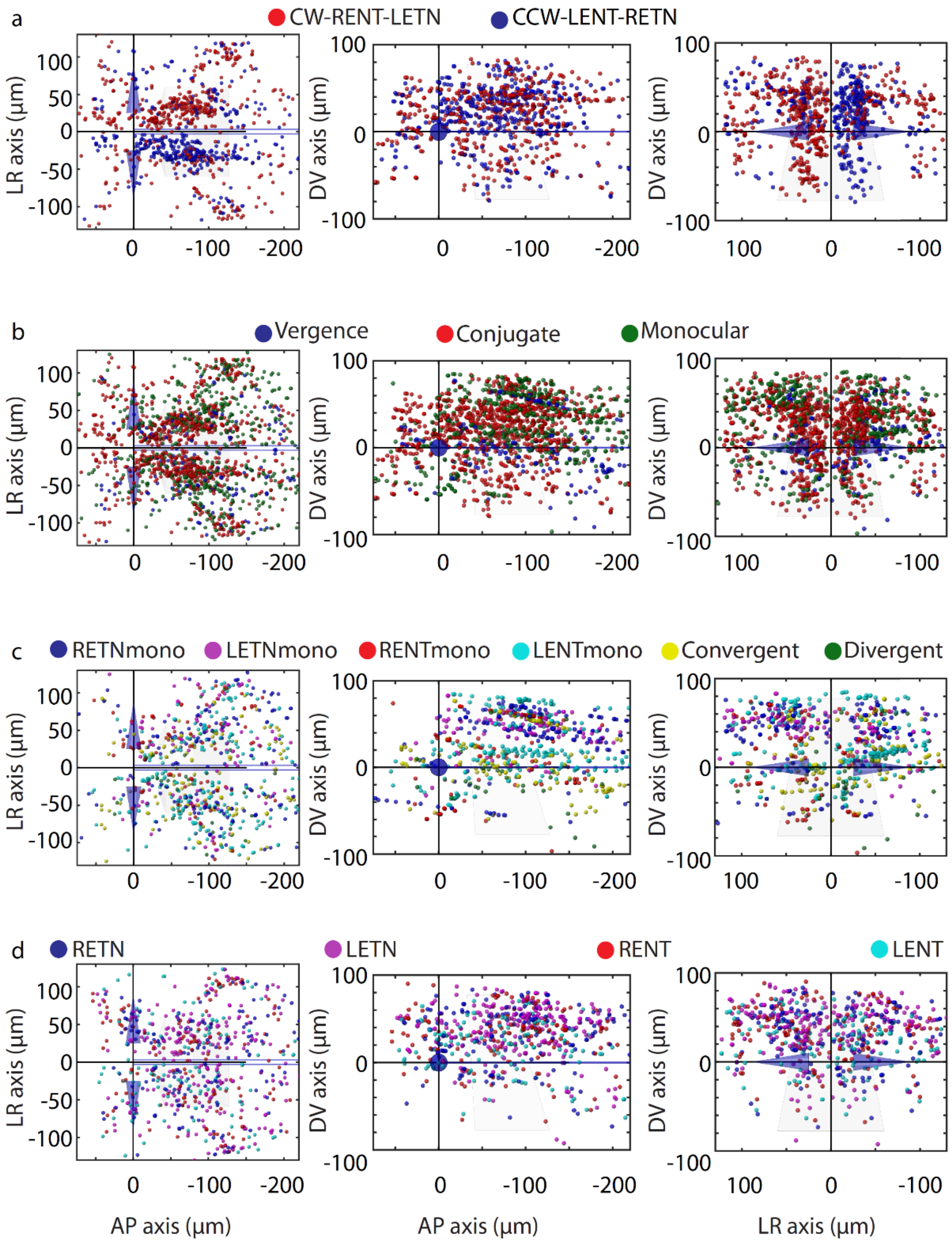

Supplementary Figure S2: Expanded cell maps. Additional anatomical cell maps showing the distribution of cell types in dorsal, sagittal and transverse planes. Each colored ball represents one neuron in one fish. The box represents the abducens nucleus, and the blue cones represent the Mauthner cell bodies. The medial longitudinal fasciculus is represented by the thick blue lines parallel to the larval midline. (a) The distribution of CW-LETN-RENT and CCW-LENT-RETN neurons. Neurons are predominantly located ipsiversively. (b) The distribution of vergence, conjugate and monocular neurons. Conjugate neurons cluster close to the midline, whereas vergence and monocular neurons

are distributed more laterally, and do not form tight clusters. (c) The distribution of all monocular neurons ($RETN_{mono}$, $LETN_{mono}$, $RENT_{mono}$, $LETN_{mono}$), convergent and divergent neurons.(d) The distribution of Helmholtz-like neurons (RETN, LETN, RENT, LENT).$RETN_{mono}$: right eye temporal nasal monocular; $LETN_{mono}$: left eye temporal nasal monocular; $RENT_{mono}$: right eye nasal temporal monocular; $LENT_{mono}$: left eye nasal temporal monocular; RETN: right eye temporal nasal; LETN: left eye temporal nasal; RENT: right eye nasal temporal; LENT: left eye nasal temporal.

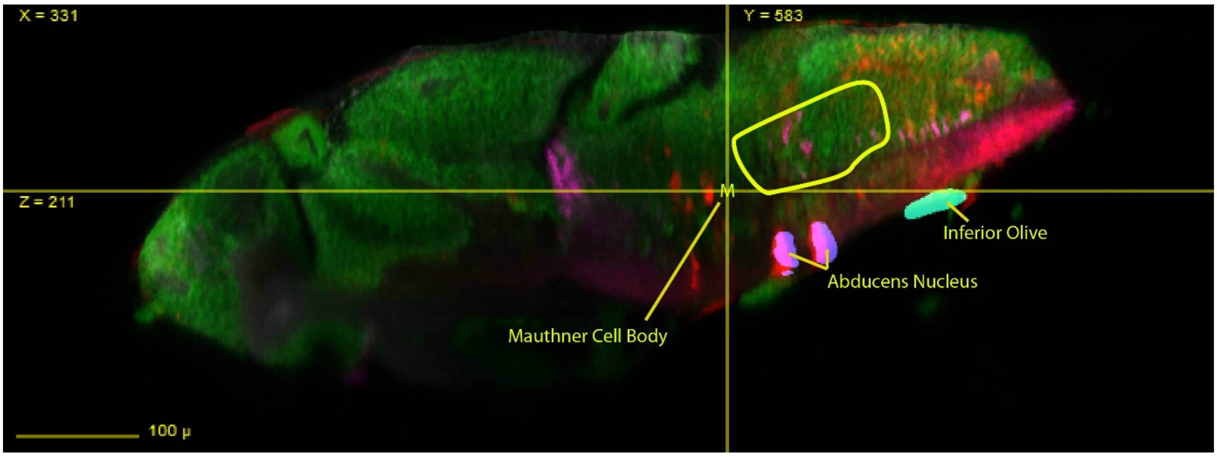

Supplementary Figure S3: Approximate location of the identified burst neuron population (yellow outline) in a reference brain using mapzebrain (Max Planck Zebrafish Brain Atlas) [1]. The x, y and z coordinates correspond approximately with the center of the Mauthner cell body. The green labelling is under control of the HuC promoter, which expresses pan-neuronally. Red shows the mnx-1 expression, which labels motoneurons. Magenta fluorescence corresponds to chx10 expression and labels descending neurons, including those involved in locomotion. This sagittal view is taken through the plane of the Mauthner cell.

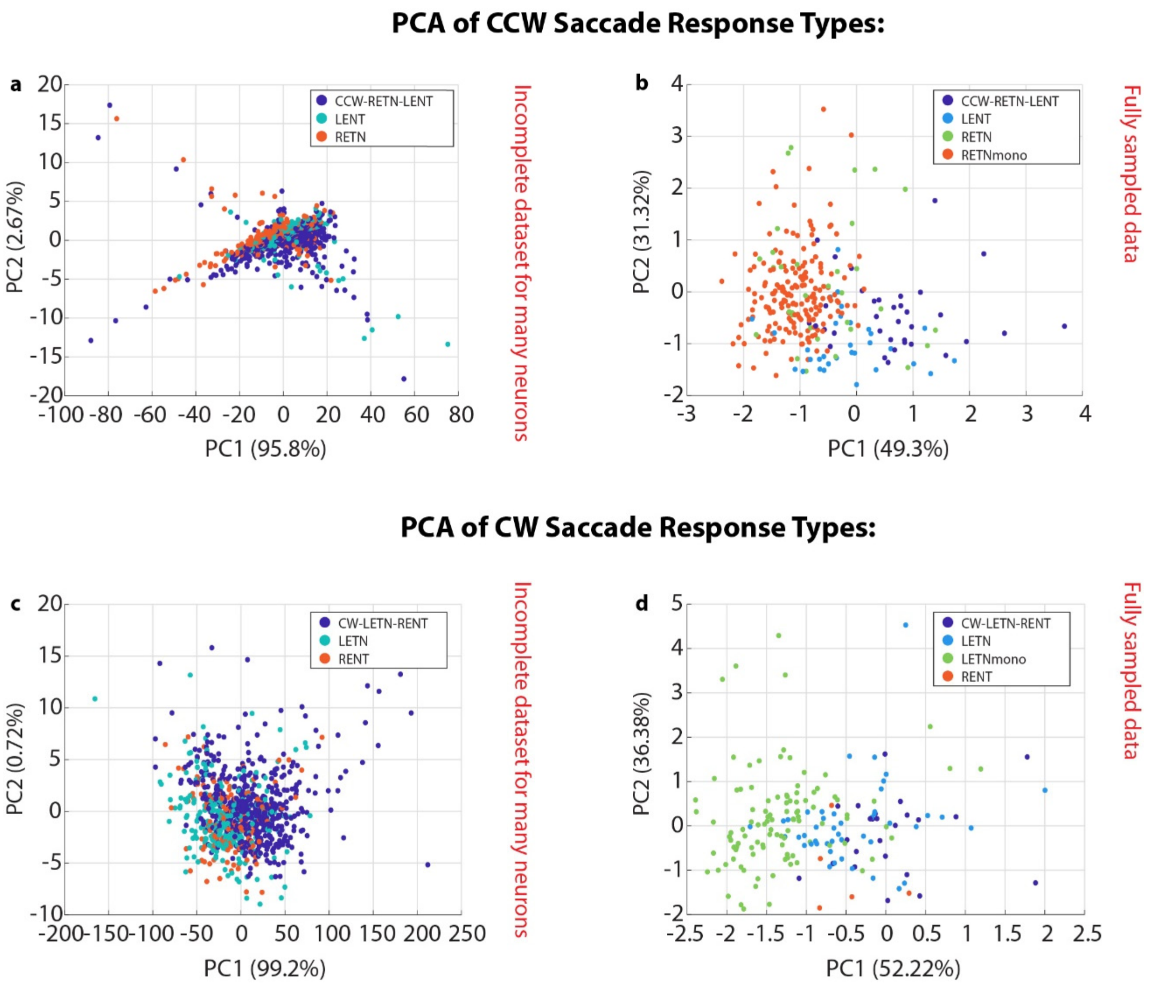

Supplementary Figure S4: Principal component analysis (PCA) of clockwise (CW) and counter-clockwise (CCW) saccade response types. PCA was carried out using the mean peri-saccadic z-score of the identified saccade-associated response types for each of the saccade types listed as dimensions, i.e. the sum of the z-score at the saccadic timepoint and the two timepoints immediately following the saccade, divided by 3. The resulting matrix size was determined as the number of neurons multiplied by the number of saccade types included (7 in b,d; 8 in a,c). The PCA analysis shown in (a) and (c) was carried out using an alternating least squares (ALS) algorithm as there were missing values in the matrices where no data was available. There were no missing values in (b) and (d), and singular value decomposition was used here. (a) CCW-RETN-LENT, LENT and RETN populations were analyzed using all unique saccade types as dimensions (CW, CCW, RETNmono, LETNmono, LENTmono, RENTmono, CONV, DIV). (b) PCA analysis was carried out on neurons belonging to the CCW-RETN-LENT, LENT, RETN and RETNmono populations, but only for neurons where the responses to the following seven saccade types were known: CW, CCW, RETN, RETNmono, LETN, LENT and RENT). (c) CCW-LETN-RENT, LETN and RENT populations were analyzed using all unique saccade types as dimensions (CW, CCW, RETNmono, LETNmono, LENTmono, RENTmono, CONV, DIV). (d) PCA analysis was carried out on neurons belonging to the CCW-RETN-LENT, LETN,

LETNmono and RENT populations where responses to the following seven saccade types were known: CW, CCW, RETN, LETN, LETNmono, LENT and RENT.

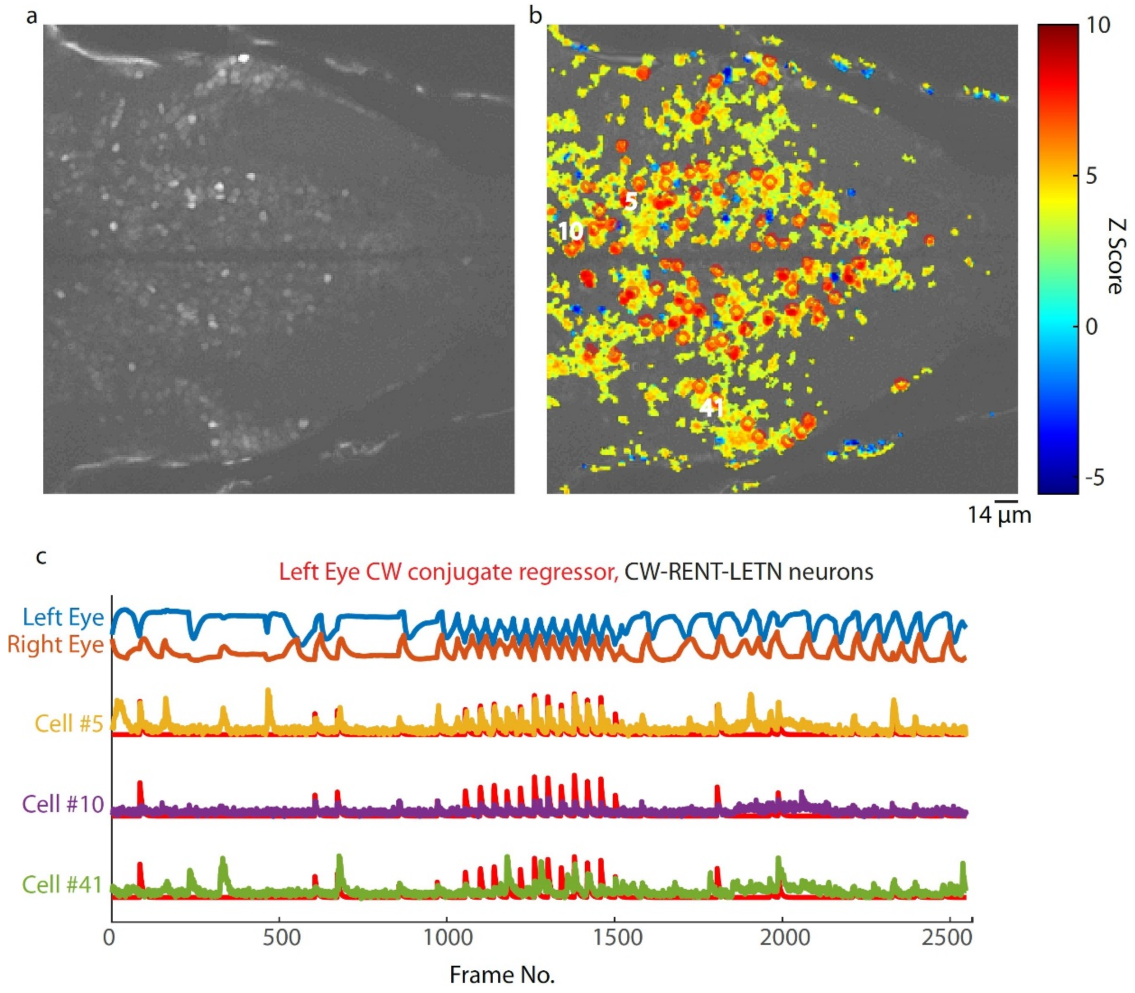

Supplementary Figure S5: Illustration of neuron identification. (a) Time-averaged image of a recorded optical slice in the in the larval zebrafish hindbrain. (b) A heat map of z-scores was generated to visualize pixels correlated with the occurrence of saccades according to the regressor analysis. Regions of interest (ROIs, shown as red circles) were manually selected. Negatively correlated pixels (blue) were not labelled due to the known properties of saccade-correlated neurons. (c) The eye traces from this recording are shown in blue and orange. The traces of three CW-LETN-RENT neurons identified in this recording are shown, alongside the CW regressor "left eye temporal-nasal conjugate" shown in red (see methods).

Supplementary Table S1: Saccade-triggered average categories.

Abbreviation	Saccade type
CW	clockwise
CCW	counter-clockwise
RENT	right eye nasal-temporal (irrespective of left eye movement)
LETN	left eye temporal-nasal (irrespective of right eye movement)
RETN	right eye temporal-nasal (irrespective of left eye movement)
LENT	left eye nasal-temporal (irrespective of right eye movement)
$RENT_{mono}$	right eye nasal-temporal monocular
$LETN_{mono}$	left eye temporal-nasal monocular
$RETN_{mono}$	right eye temporal-nasal monocular
$LENT_{mono}$	left eye nasal-temporal monocular
CONV	convergent
DIV	divergent

Supplementary Table S2: Expanded list of classified neuron types. Identified neuron types are sorted according to the number of neurons for each type. Neuron types for which more than one neuron has been found are included in this table.

**Rank**	Neuron Type	Number of Neurons
**1**	' CW LETN RENT'	517
**2**	' CCW RETN LENT'	442
**3**	' LENTmono'	266
**4**	' LETN'	262
**5**	' RETNmono'	184
**6**	' RETN'	180
**7**	' convergent'	150
**8**	' RENT'	144
**9**	' LENT'	133
**10**	' LETNmono'	108
**11**	' CCW LENT'	106
**12**	' RENTmono'	104
**13**	' CW LETN'	99
**14**	' CW CCW RETN LETN LENT RENT'	98
**15**	' RETN LETN'	96
**16**	' RETN RETNmono'	95
**17**	' LETN RENT'	93
**18**	' LETN LETNmono'	92
**19**	' CW RENT'	89
**20**	' CCW RETN'	84
**21**	' CCW'	79
**22**	' RETN LENT'	64
**23**	' RETN LETN RENT'	53
**24**	' CW'	45
**25**	' divergent'	35
**26**	' CW LETN LETNmono RENT'	31
**27**	' CW RETN LETN RENT'	30
**28**	' CCW RETN LETN LENT'	26
**29**	' LENT LENTmono'	20
**30**	' RETN RETNmono LENT'	19
**31**	' RETN LETN LENT RENT'	19
**32**	' RETN LETN LENT'	18
**33**	' RENT RENTmono'	18
**34**	' CCW RETN LENT convergent'	17
**35**	' CW LETN RENT RENTmono'	14
**36**	' LETN convergent'	14
**37**	' CW RETN LETN LENT RENT'	13
**38**	' CCW RETN LETN LENT RENT'	13

**39**	' CCW RETN LENT divergent'	13
**40**	' RETN LETN LETNmono'	13
**41**	' RETN convergent'	13
**42**	' CCW RETN RETNmono LENT'	12
**43**	' RETNmono LETN'	11
**44**	' LETN LETNmono RENT'	11
**45**	' LETNmono convergent'	11
**46**	' CW RETN LETN LETNmono RENT'	10
**47**	' RETN RENT'	10
**48**	' CCW RETN RETNmono'	9
**49**	' CCW RETN LENT LENTmono'	9
**50**	' RETN RETNmono LETN'	9
**51**	' LETN LETNmono convergent'	9
**52**	' LETN RENTmono convergent'	9
**53**	' LENT divergent'	9
**54**	' CW LETN LENT RENT'	8
**55**	' CCW RETN RETNmono LETN LENT convergent'	8
**56**	' CW CCW LETN LENT RENT'	7
**57**	' CCW RETN LETNmono LENT'	7
**58**	' CCW LENT divergent'	7
**59**	' LENT RENT'	7
**60**	' CW CCW RETN LETN LETNmono LENT RENT'	6
**61**	' CW CCW RETN LENT'	6
**62**	' RETNmono LETN LETNmono'	6
**63**	' RETNmono LETNmono'	6
**64**	' CW CCW RETN LENT RENT'	5
**65**	' CW RENT RENTmono'	5
**66**	' CCW RETN LENT LENTmono convergent'	5
**67**	' CCW RETN LENT RENT'	5
**68**	' CCW LENT LENTmono'	5
**69**	' RETN LETN convergent'	5
**70**	' RETN LETNmono'	5
**71**	' LETN RENT RENTmono convergent'	5
**72**	' LETN RENT RENTmono'	5
**73**	' LENT LENTmono divergent'	5
**74**	' CW CCW RETN LETN LENT'	4
**75**	' CCW RETN LETN LETNmono LENT'	4
**76**	' CCW RETN LETN RENT'	4
**77**	' CCW RETN LETN'	4
**78**	' CCW RETN LENT convergent divergent'	4
**79**	' RETN RETNmono LENT LENTmono'	4
**80**	' RETN LENT RENT'	4
**81**	' RETN LENT convergent'	4
**82**	' RETNmono LETN RENT'	4
**83**	' RETNmono LENT'	4
**84**	' CW CCW RETN LETN LENT RENT convergent'	3

**85**	' CW RETN LETN'	3
**86**	' CW LETN RENT convergent'	3
**87**	' CCW RETN RETNmono LETN LETNmono LENT convergent'	3
**88**	' CCW RETN LETN LENT LENTmono convergent'	3
**89**	' CCW RETN LETN LENT RENT convergent'	3
**90**	' CCW RETN LETN LENT convergent'	3
**91**	' CCW RETN LENT RENTmono'	3
**92**	' CCW RETN convergent'	3
**93**	' CCW LENT LENTmono RENT'	3
**94**	' RETN LETNmono convergent'	3
**95**	' RETN LENT LENTmono'	3
**96**	' RETN LENT convergent divergent'	3
**97**	' RETN LENT divergent'	3
**98**	' RETNmono LENTmono'	3
**99**	' LETN LENT'	3
**100**	' LETNmono LENTmono'	3
**101**	' LETNmono RENT'	3
**102**	' RENT RENTmono convergent'	3
**103**	' CW CCW RETN RETNmono LETN LENT RENT'	2
**104**	' CW CCW RETN LETN LENT LENTmono RENT'	2
**105**	' CW CCW RETN LETN RENT'	2
**106**	' CW CCW RETN LETN'	2
**107**	' CW CCW RETNmono LENT'	2
**108**	' CW CCW LETN RENT'	2
**109**	' CW LETN LENT LENTmono RENT'	2
**110**	' CW LETN RENT divergent'	2
**111**	' CW LENT RENT'	2
**112**	' CCW RETN RETNmono LETN LENT'	2
**113**	' CCW RETN RETNmono LENT convergent'	2
**114**	' CCW RETN LETN LETNmono'	2
**115**	' CCW RETN LETN LENT LENTmono'	2
**116**	' CCW RETN LETN LENT RENT RENTmono'	2
**117**	' CCW RETN LETN LENT RENTmono'	2
**118**	' CCW RETN LETN LENTmono RENT convergent'	2
**119**	' CCW RETN LENT LENTmono RENT'	2
**120**	' CCW RETN convergent divergent'	2
**121**	' CCW RETNmono LENT'	2
**122**	' CCW LETN LENT'	2
**123**	' CCW LETN'	2
**124**	' CCW LENT RENT'	2
**125**	' RETN RETNmono LETN LETNmono convergent'	2
**126**	' RETN RETNmono LETNmono convergent'	2
**127**	' RETN RETNmono LETNmono'	2
**128**	' RETN LETN LENT LENTmono convergent'	2
**129**	' RETN LETN LENT convergent'	2

**130**	' RETN LENTmono convergent'	2
**131**	' RETN RENTmono'	2
**132**	' LETN RENTmono'	2
**133**	' LENT LENTmono RENT RENTmono divergent'	2
**134**	' LENTmono RENTmono'	2
**135**	' RENT convergent'	2
**136**	' RENTmono convergent'	2

References

1. Kunst, M., et al., *A Cellular-Resolution Atlas of the Larval Zebrafish Brain.* Neuron, 2019. **103**(1): p. 21-38.e5.
